# Does the presence of blind-ended vas deferens and spermatic vessels in laparoscopic exploration of non-palpable testes conclusively indicate testicular absence?

**DOI:** 10.3389/fped.2024.1413099

**Published:** 2024-06-18

**Authors:** Chang-Kun Mao, Qi-Fei Deng, Xiang Liu, Yong-Sheng Cao, Guang-Yuan Li

**Affiliations:** ^1^ Department of Urology, Lu’an People’s Hospital of Anhui Province, Lu’an Hospital of Anhui Medical University, Lu’an, Anhui, China; ^2^ Department of Urology, Anhui Provincial Children’s Hospital Affiliated of Anhui Medical University, Hefei, Anhui, China

**Keywords:** non-palpable testis, cryptorchidism, children, laparoscopic exploration, testicular atrophy

## Abstract

**Objective:**

The purpose of this study was to determine whether the presence of blind-ended vas deferens and spermatic vessels (VDSV) during laparoscopic exploration of non-palpable testes (NPT) indicates testicular absence or atrophy.

**Materials and methods:**

A retrospective analysis was conducted on clinical data of patients diagnosed with NPT and treated with surgical intervention at our center from April 2013–April 2023. The dataset encompassed information such as the children's age, affected side, size of the contralateral testis, surgical procedures employed, outcomes, and histopathological examination results. All patients underwent physical examination and ultrasonography preoperatively, followed by a combination of laparoscopic exploration and exploration through inguinal or scrotal incisions during surgery. Long-term follow-up was conducted postoperatively.

**Results:**

A total of 476 cases comprising 504 NPT were included in this study: 302 cases on the left side, 146 cases on the right side, and 28 cases bilaterally. All patients underwent surgical treatment within 6–126 months (median 13 months). During laparoscopic exploration, blind-ended VDSV were found in 90 testes (72 on the left side, 18 on the right side), while exploration through inguinal or scrotal incisions revealed 52 (57.8%) testicular nodules with atrophy, which were excised, leaving 38 (42.2%) without any findings. Histopathological examination of atrophic nodules revealed fibrosis as the most common finding in 41 cases (78.8%), followed by involvement of the vas deferens in 33 cases (63.5%), calcification in 24 cases (46.2%), epididymis in 23 cases (44.2%), and hemosiderin deposition in 7 cases (13.6%). Fibrosis, calcification, hemosiderin deposition, involvement of the vas deferens, and epididymis were found in combination in 47 specimens (90.4%). Seminiferous tubules (SNT) were found in 3 specimens (5.7%), and germ cells (GC) were found in 1 specimen (1.9%).

**Conclusion:**

The presence of blind-ended VDSV during laparoscopic exploration of NPT does not necessarily indicate testicular absence or disappearance. It is possible that atrophic testicular nodules are located within the inguinal canal or scrotum. This understanding contributes to the management of non-palpable testes. Considering their unpredictable malignant potential, we recommend excision.

## 1 Introduction

Full-term and preterm newborns can both experience cryptorchidism, but the incidence varies and depends on gestational age. The incidence of cryptorchidism in full-term infants is approximately 1.0%–4.6%, while in preterm newborns, it can reach up to 1.1%–45% (1). Currently, the most clinically useful classification categorizes cryptorchidism into palpable and non-palpable testes (NPT), with around 15%–24% of cryptorchidism cases being non-palpable (1–4). Whether the testis is palpable determines the choice of surgical approach and technique. European guidelines currently classify NPT as intra-abdominal cryptorchidism, inguinal cryptorchidism, testicular absence, and sometimes include some cases of ectopic testes (1).

In clinical practice, if laparoscopic exploration reveals that the vas deferens and spermatic vessels (VDSV) end blindly before entering the internal ring, it suggests testicular absence or disappearance, eliminating the need for further exploration (1, 5, 6). However, in our clinical experience, we have found that this is not always the case. Laparoscopic exploration may show blind-ended VDSV before entering the internal ring, but exploration through groin or scrotal incisions may reveal the presence of atrophic testicular nodules or poorly developed testes in some patients. Such cases are relatively rare in the literature, and there is controversy regarding the surgical management (7), prompting us to review the case data of NPT in our center over the past 10 years. This has led us to reevaluate whether the blind-ended appearance of VDSV before entering the internal ring truly indicates testicular absence or disappearance, or if atrophic testicular nodules exist in the groin or scrotum.

## 2 Methods

### 2.1 Selection criteria

We retrospectively collected clinical data of 8,827 cases of cryptorchidism who underwent surgical treatment in the Department of Urology at the Affiliated Lu'an Hospital and Affiliated Children's Hospital of Anhui Medical University from April 2013–April 2023 through the electronic medical records system. Among them, 476 cases (5.4%) involving a total of 504 testes were diagnosed as non-palpable. The diagnosis of NPT was made by the attending physician in the outpatient clinic and was defined as the inability to palpate the testis in the inguinal or scrotal area of the child while in a supine or squatting position in a warm environment, and also the ultrasound examination could not detect the testis (8). Prior to the start of the surgical exploration, a re-examination of the child was conducted under general anesthesia through palpation. This re-examination may reveal that some children have palpable testes, in which case these children were excluded from this study. Palpable testes can assist surgeons in selecting the correct surgical site and technique, thereby avoiding unnecessary laparoscopic exploration.

Detailed records were kept of the children's physical examinations, ultrasound examination results, and surgical data. To determine if the contralateral testis was enlarged, we measured the maximum longitudinal diameter of the contralateral testis by ultrasound. We used a threshold of 16 millimeters as the definition of enlargement for children under 36 months (9, 10). This study obtained approval from the Ethics Committees of Anhui Medical University Affiliated Lu'an Hospital (Approval No.A20230620) and the Affiliated Children's Hospital (Approval No.116123S28).

### 2.2 Surgical management

All children with NPT underwent surgical exploration, with ages ranging from 6–126 months (median age 13 months). Initially, all children underwent laparoscopic exploration to determine whether the testis was located inside the abdomen. If the exploration revealed blind-ended VDSV before entering the internal ring, groin or scrotal exploration was performed to confirm the presence of atrophic testicular nodules. If atrophic testicular nodules were present, they were excised and sent for histopathological examination.

### 2.3 Statistical analysis

Statistical analyses were performed using SPSS Statistics, version 25.0 (IBM Corp., Armonk, NY, United States). Continuous variables were presented as mean ± standard deviation (SD) or mean (min-max), and categorical variables were presented as *n*(%). Comparisons of continuous variables were made using independent samples *t*-tests, while comparisons of categorical variables were made using chi-squared or Fisher's exact tests. All tests were two-tailed. A significance level of *P* < 0.05 was considered statistically significant.

## 3 Results

**Figure 1:**
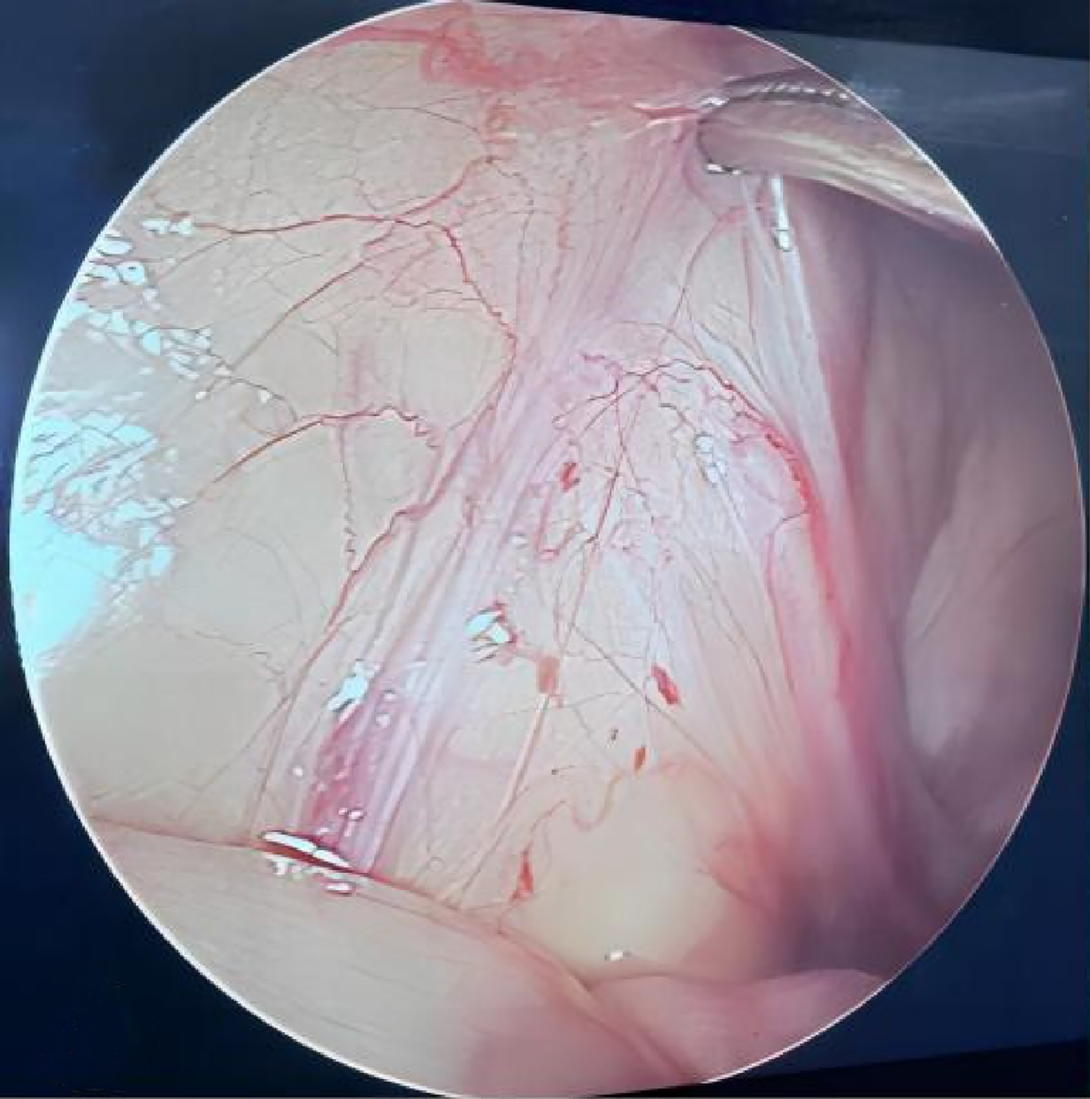
During laparoscopic examination, the vas deferens and spermatic vessels ended blindly before entering the closed internal ring.

This study included a total of 476 cases comprising 504 NPT: 302 on the left side, 146 on the right side, and 28 bilateral cases. Laparoscopic exploration revealed that 248 testes had spermatic vessels entering a closed internal ring, 118 were intra-abdominal testes, and 48 were peeping testes. The remaining 90 testes, where the blind-ended VDSV terminated before reaching the internal ring (Figure 1), constituted our analysis sequence. Exploration through inguinal or scrotal incisions revealed 52 (52/90, 57.8%) atrophic testicular nodules, which were excised and sent for histopathological examination, while the remaining 38 (38/90, 42.2%) did not reveal any findings (Table 1). Among these 90 testes, preoperative color Doppler ultrasound examination revealed 23 atrophic nodules, with 7 located in the inguinal region and 16 within the scrotum. We confirmed the presence of these nodules intraoperatively. Among the 58 patients aged less than 36 months, 38 cases (65.5%) exhibited contralateral testicular enlargement (CTE), with ultrasound examination revealing a longitudinal diameter greater than 16 millimeters. Comparison between the two groups revealed a statistically significant difference in preoperative ultrasound findings (*P *< 0.05), while no significant differences were found in the age of the patients, affected side, CTE, and size of the contralateral testicle (*P *> 0.05). Interestingly, we observed a fourfold higher incidence of this condition on the left side compared to the right side. Furthermore, we noted that the blood supply to these 52 atrophic testicular nodules originated from the tissues within the scrotum rather than from the spermatic cord and vas deferens vessels coming from the abdominal direction.

**Table 1:** Comparison of inguinal or scrotal exploration results of blind-ended VDSV after laparoscopic examination.

Factors	Testicular nodules	No findings	*t* (*x*^2^) value	*P-*value
	(*n* = 52)	(*n* = 38)		
Age at surgery(m)				
median ± SD	13.6 ± 2.5	15.2 ± 3.4	0.325	0.751
Affected side (*n*, %)			0.729	0.393
Left	40 (76.92%)	32 (84.21%)		
Right	12 (23.08%)	6 (15.79%)		
CTE(Age < 36 m, *n*, %)			0.288	0.592
Yes	20 (38.46%)	18 (47.39%)		
No	12 (23.08%)	8 (21.05%)		
Contralateral testicular size(millimeters)				
median ± SD	26.8 ± 8.5	28.4 ± 9.6	0.819	0.415
Preoperative US findings			25.346	0.000^a,b^
Inguinal region	7 (13.46%)	0 (0%)		
Scrotum	16 (30.77%)	0 (0%)		
No findings	29 (55.77%)	38 (100%)		
Intraoperative nodules location			-	-
Inguinal region	35 (67.31%)	0 (0%)		
Scrotum	17 (32.69%)	0(0%)		

^a^ Fisher's exact test.

^b^ *P *< 0.05.

**Figure 2:**
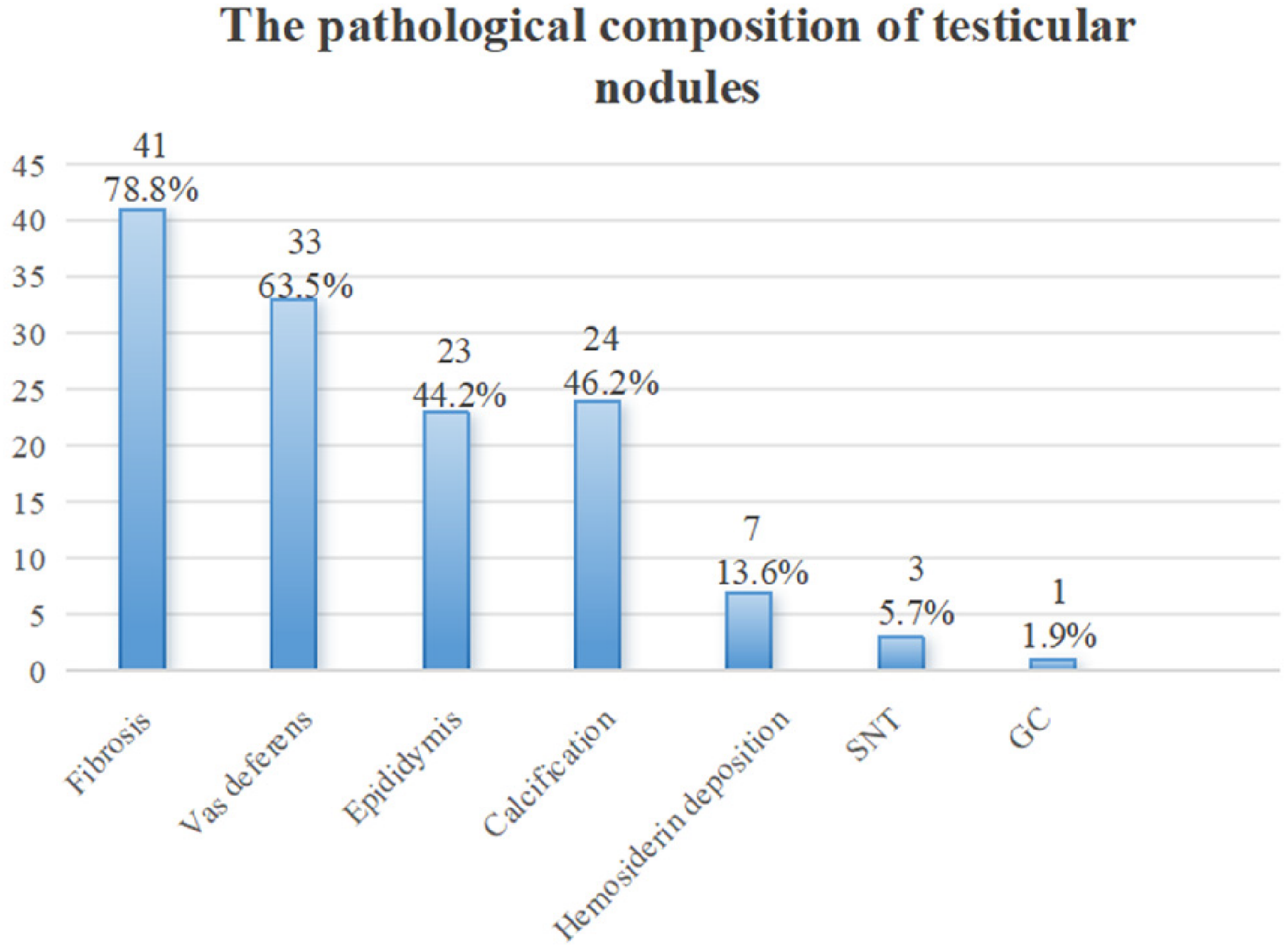
The pathological composition of testicular nodules.

The results of hematoxylin and eosin (H&E) staining of excised atrophic nodules showed that fibrosis was the most common finding in 41 cases (78.8%), followed by involvement of the vas deferens in 33 cases (63.5%), calcification in 24 cases (46.2%), epididymis in 23 cases (44.2%), and hemosiderin deposition in 7 cases (13.6%). Among 47 specimens (90.4%), a combination of fibrosis, calcification, hemosiderin deposition, involvement of the vas deferens, and epididymis was observed. Seminiferous tubules (SNT) were found in 3 tissues (5.7%), and germ cells (GC) were found in 1 tissue (1.9%) (Figure 2).

Regular outpatient follow-up examinations were conducted postoperatively, including physical examination and ultrasound examination. Follow-up intervals ranged from 3 months to 1 year, with an average follow-up time of 40 months (range, 3–108). All 90 patients showed good wound healing without complications such as infection. The contralateral testis exhibited normal development, and there were no occurrences of testicular torsion.

## 4 Discussion

Preoperative diagnosis of cryptorchidism primarily relies on physical examination and auxiliary tests, and it is essential to accurately determine the presence of the testis before surgery. Currently, ultrasonography is one of the important auxiliary tools (11). Through ultrasonic imaging, physicians can clearly observe the position, size, and morphology of the testis. This helps to identify the location of palpable testes such as in the inguinal canal and assists surgeons in selecting appropriate surgical procedures. However, NPT are often located intra-abdominally or are absent (12). Different imaging modalities, such as ultrasonography (13) or MRI (14), have limited efficiency in these cases. Although ultrasound is non-invasive, it is time-consuming, expensive, and lacks accuracy in detecting the presence of the testis or determining the absence of intra-abdominal testes when the testis cannot be palpated. Therefore, both the American Urological Association (AUA) and the European Association of Urology/European Society for Pediatric Urology (EAU/ESPU) do not recommend the use of ultrasonography in the management of NPT (1, 15). However, in another survey, more European doctors (49%) still chose ultrasonography as the first management step for NPT compared to American surgeons (12%) (16). Press et al. (17) conducted color Doppler ultrasonography on 31 patients with NPT, and the results showed that 87.5% of NPT avoided laparoscopic surgery after feasible testes were identified by ultrasound. In our institution, all NPT are routinely examined by ultrasonographers, and the examination cost is relatively low (approximately $13). In our study cohort, ultrasound detected atrophic testicular nodules in 44.23% (23/52) of cases within the inguinal or scrotal regions. Therefore, we believe that ultrasound examination is useful in the management of NPT. Another important step in the management of NPT is to conduct a thorough re-examination of the patient after general anesthesia administration, as testes that were not palpable preoperatively may become palpable when the patient is under general anesthesia (1). Otherwise, the simplest and most accurate method for the diagnosis and treatment of NPT is laparoscopic exploration (1, 15, 18). Laparoscopic exploration of NPT can reveal various possibilities, including spermatic vessels entering the internal ring (40%), intra-abdominal (40%), or peeping (10%) testes, or confirmation of testicular absence when the VDSV end blindly (19). In our study of 504 NPT, blind-ended VDSV were observed in 90 (17.9%) cases during laparoscopic exploration, consistent with the reported literature.

Laparoscopic exploration not only assists in the localization and diagnosis of NPT but is also considered the optimal approach for surgical treatment of intra-abdominal undescended testes (20–22). However, there is controversy regarding laparoscopic exploration as the initial treatment approach. Igarashi et al. (8) argue that the incidence of intra-abdominal testes is lower than that of inguinal or absent testes, suggesting that inguinal exploration should be the initial surgical method for NPT. One clinical symptom that aids in determining the primary surgical approach is CTE (9, 23). If the contralateral testis measures over 16–20 mm in length, the presence of a solitary testis is highly probable, and inguinal incision may be chosen as the primary method with excision of atrophic testicular nodules (10). However, if no residual testicular remnants or blind-ending vas deferens are found in the inguinal canal or scrotum, laparoscopic examination is warranted. Another survey targeting pediatric surgeons in the United States and Europe showed that regardless of contralateral testicular size, 81%–97% of surgeons chose laparoscopic exploration as the initial step. However, in the presence of CTE, 7%–11% of surgeons indeed changed their choice from diagnostic laparoscopic exploration to open inguinal exploration (16). Among the 90 patients with blind-ended vessels in this study, 58 patients were younger than 36 months, and 38 cases (65.5%) exhibited CTE, with ultrasound examination revealing a longitudinal diameter greater than 16 millimeters. Therefore, we believe that for patients with compensatory CTE, considering the higher possibility of extra-abdominal atrophic testes, inguinal incision exploration should be preferred in subsequent surgical procedures, potentially avoiding unnecessary laparoscopic exploration (24).

Laparoscopic exploration revealing blind-ended VDSV before entering the internal ring suggests testicular absence or disappearance, thus obviating the necessity for further exploration in the inguinal canal or scrotum, which is the current consensus (1, 6, 15). However, Mah et al. (16) reported that considering the possibility of testicular nodules in the inguinal canal or scrotum, 17% of American and European surgeons performed inguinal exploration despite the presence of blind-ended vessels. Interestingly, in our study, even when laparoscopic exploration indicated blind-ended VDSV before entering the internal ring, inguinal exploration revealed atrophic testicular nodules (52/90, 57.8%). This finding is perplexing since traditionally, the testis is assumed to be connected to the VDSV. Moreover, we also observed that the blood supply to these testicular nodules originated from the tissues within the scrotum, rather than from the spermatic vessels and vas deferens originating from the abdominal direction. We speculate that the possible reason could be testicular torsion occurring after the testis had descended into the inguinal canal or scrotum during late pregnancy (25). This hypothesis is supported by the presence of hemosiderin-laden macrophages in the surgically excised specimens (26), consistent with venous congestion and hemorrhagic infarction secondary to torsion. The prevalence and support for this theory were widespread in our study, as evidenced by the presence of fibrosis, dystrophic calcification, hemosiderin deposition in vascular accidents, and macrophages in excision residues (27). Interestingly, we observed that this phenomenon occurred four times more frequently on the left side than on the right, possibly because the left testis descends earlier than the right (26). Further exploration is warranted to elucidate the specific reasons.

There is ongoing debate regarding the necessity of excising atrophic testicular nodules. Woodford et al. (12) evaluated the histopathological results of 30 excised testicular nodules and found no evidence of viable germ cells (GC). Therefore, they proposed that excision of testicular nodules is unnecessary based on the malignant potential of residual testicular tissue, a sentiment echoed by other scholars (28, 29). A recent systematic review indicated that the overall incidence of GC was 5.3%, and the overall incidence of seminiferous tubules (SNT) was 10.7% (30). The low incidence of GC and the minimal malignant potential lean towards arguing against the necessity of excision. However, this overlooks the surgical practice aspect from our perspective. The primary objective of surgery for NPT exploration is to determine the presence of viable testes, which can only be achieved by exposing the terminal end of the VDSV, with excision not adding significantly to the surgical time in such cases (31). On the other hand, considering the potential malignant precursor in atrophic nodules, most surgeons prefer excising the nodules during exploration, with laparoscopic inguinal exploration serving as a good alternative to open inguinal exploration (32). In line with our study, where laparoscopic exploration revealed blind-ended VDSV, we proceeded with inguinal exploration and excision of atrophic testicular nodules, whereas previous extensive research (1, 5, 6, 15) classified this condition as testicular absence, thereby not exploring further. Here, we arrive at a different diagnosis—testicular atrophic nodules rather than testicular absence. Such a diagnosis facilitates effective communication with caregivers and subsequent follow-up observations.

This study has several limitations. Firstly, as a retrospective study, it is subject to inherent limitations such as the completeness of data and selection bias. Secondly, in this study, all testicular nodules were excised, and the lack of a control group with preserved nodules prevents us from assessing their potential future malignancy risk. Additionally, the variability in surgical techniques and the experience of the surgeons involved could influence the results.

## 5 Conclusion

The presence of blind-ended VDSV during laparoscopic exploration for NPT does not definitively indicate testicular absence or disappearance. It is possible that atrophic testicular nodules are located within the inguinal canal or scrotum. If the surgeon's goal is to excise all nodules, regardless of the appearance of vessels during laparoscopic examination, open exploration is warranted. This understanding aids in the management of NPT, considering their unpredictable malignant potential. Therefore, we recommend excision.

## Data Availability

The raw data supporting the conclusions of this article will be made available by the authors, without undue reservation.
